# Effect of intrauterine injection of human chorionic gonadotropin before embryo transfer on clinical pregnancy rates from *in vitro* fertilisation cycles: a prospective study

**DOI:** 10.1186/1477-7827-12-9

**Published:** 2014-01-29

**Authors:** Álvaro Santibañez, Jorge García, Olga Pashkova, Omar Colín, Guillermo Castellanos, Ana P Sánchez, Julio F De la Jara

**Affiliations:** 1 Procrea, Reproductive Centre, 1st floor 670 Ejercito Nacional Avenue, Polanco Reforma, 11550 Mexico City, Mexico

**Keywords:** Intrauterine hCG, Implantation rate, Pregnancy rate, IVF, ICSI

## Abstract

**Background:**

The implantation process after embryo transfer depends on the embryo quality and endometrial receptivity. It is estimated that fifty to seventy-five per cent of pregnancies are lost due to a failure of implantation. There is evidence that there is an early secretion of human chorionic gonadotrophin before embryo implantation, and this secretion has been linked to an important function in angiogenesis and the inflammatory response that promotes the implantation process. Our objective was to determine the effects of intrauterine injection of human chorionic gonadotropin (hCG) before the embryo transfer in an *in vitro* fertilisation cycle.

**Methods:**

A prospective randomised study was conducted in Reproductive Medicine Centre PROCREA in Mexico City. Infertile patients who had a medical indication for *in vitro* fertilisation were studied. Two groups were included (n 210); the intervention group received an intrauterine injection of 500 IU of hCG before the embryo transfer (n 101). The control group (n 109) did not receive hCG. Comparisons were performed using a chi-square test.

**Results:**

The clinical pregnancy rate (CPR) was our principal outcome. The implantation rate was a secondary outcome. The implantation rate was significantly higher in the hCG group compared to the control group (52.4% vs 35.7%, p 0.014). The clinical pregnancy rate was also significantly higher (50.4 vs 33.0%, p 0.010). No adverse effects were observed.

**Conclusions:**

The intrauterine injection of hCG before embryo transfer showed a significant increase in the clinical pregnancy rate. More clinical trials are needed to reproduce these results on this promising intervention. The live birth rate must be included in subsequent studies.

## Background

Infertility is defined as the failure to conceive after 12 months or more of regular intercourse without contraception. Epidemiological studies have shown that 80% of the couples had conceived during that period. It is estimated that 15% of the couples are infertile in developed countries [1]. There has not been changes in prevalence; nevertheless, there has been reported a substantial increase in demand for the treatments [2]. *In Vitro* Fertilisation (IVF) is a highly complex technique that involves the use of standardised protocols for a controlled ovarian stimulation, oocyte retrieval under ultrasound guidance, fertilisation of gametes in the laboratory, embryo culturing and embryo transfer [2]. The American Society for Reproductive Medicine (ASRM) and the Society for Assisted Reproductive Technology Registry (SART) report annually the live birth rates. For *in vitro* fertilisation cycles, the success rate reported is 40.1% in women less than 35 years to 20.6% in women in the 41–42 year range [3]. The implantation process after the embryo transfer depends on the embryo quality and the endometrial receptivity. It is estimated that approximately 50% to 75% of lost pregnancies are due to a failure of implantation [4].

Human chorionic gonadotropin (hCG) is a heterodimeric placental glycoprotein hormone that is required to maintain pregnancy. The hCG is initially produced by the blastocyst 6–8 days after fertilisation [5,6]. There is evidence of an early secretion of human chorionic gonadotropin by embryos of primates before implantation. Different mechanisms have been described in which hCG can regulate implantation. An *in vitro* study demonstrated that hCG is a potent attractor of inflammatory cells, such as neutrophils, monocytes, and lymphocytes [7]. The hCG directly regulates endothelial cell responsiveness to interleukin 1 and amplifies the cytokine-mediated effect on cell proliferation, migration and release of angiogenic factors [8]. Embryo implantation requires an extensive angiogenesis at the maternal-fetal interface. The hCG can modulate the receptivity of the endometrial stromal cells to interleukin-1 by upregulating its receptor (IL1R) during the implantation window. This function has an impact on angiogenesis, which is a pathway by which embryonic growth is promoted [9]. Berndt et al. reported that hCG displayed a potent angiogenic effect through receptor activation of transforming growth factor beta (TGF-β) in endothelial cells, which is a key role in placental development [10]. The control of complementary activation in the embryo environment has been demonstrated to be critical for embryo survival. Palomino et al. evaluated the expression of complementary proteins in response to the hCG. They found an increase in the C3 protein mRNA levels in stromal cells and an increase in the decay-accelerating factor in epithelial cells of an endometrial biopsy culture of infertile women during the implantation window [11]. Litch et al. developed an intrauterine microdialysis device to measure paracrine mediators. After the administration of 500 UI of hCG, they found a significant inhibition of intrauterine insulin-like growth factor binding protein 1 (IGFBP-1) and the macrophage colony-stimulating factor (M-CSF), while leukemia inhibitory factor (LIF), the vascular endothelial growth factor (VEGF) and the matrix metallopeptidase 9 (MMP-9) were significantly stimulated. These multiple effects appear to precede the classical endocrine role of the hCG and could be directly involved in the regulation of embryo implantation [12].

These mechanisms have been used to design clinical studies to assess clinical outcomes upon implantation. Xiao-Yan et al. measured human embryonic human chorionic gonadotropin in embryo culture media that was used during *in vitro* fertilisation (IVF) cycles, and they found a positive correlation between the beta-hCG concentration and the implantation rate, which indicates that the hCG that is secreted by embryos could be used as a biomarker for embryo selection in IVF cycles [13]. Mansour et al. reported the first time use of an intrauterine injection of hCG before the embryo transfers, and they found a significant improvement in the pregnancy rates of the IVF cycles [14]. These improvements could be explained by various changes that are produced in the endometrium, which is where human chorionic gonadotropin has an effect on implantation [5-12].

Our objective was to reproduce and confirm the benefits to the clinical effects of intrauterine injection of hCG before embryo transfer on the pregnancy rates in the IVF cycles at our reproductive centre.

## Methods

This study was a prospective observational study that was approved by the internal board and ethical committee. A total of 210 infertile women under the age of 40 who had an indication for an IVF/ICSI cycle were enrolled in the study. The exclusion criterion was azoospermia. The study subjects were recruited voluntarily. A simple randomisation sample and assignment was generated in a computer-based program. All of the patients were counselled, and a written informed consent was obtained from all of the participants in this study. The screening process for enrolment was conducted during scheduled medical appointments in the PROCREA reproductive centre, and the patients were followed until a pregnancy test; if the pregnancy test was positive, then the pregnancy outcomes were followed. The data collection was prolective. We observed two groups; one group (n 101) was injected with 500 UI of intrauterine hCG before embryo transfer. The second group (n 109) did not receive the administration of hCG. The ovarian stimulation regimen chosen was an uncontrolled variable because the regimen chosen was indicated based on individual patient characteristics. Consecutive patients were assigned with simple randomisation. The preparation of the intrauterine injection of hCG that is described by Mansour’s technique consisted of adding 500 UI of hCG (Choragon, Ferring Pharmaceuticals) to 1 mL of culture media (G2, Vitrolife). Our dilution was prepared to obtain a dose of 500 UI of hCG with the lowest level of medium. The hCG for intrauterine injection was prepared by adding 0.2 mL of tissue culture media (G2) to one vial that contained 5,000 IU of hCG. Fecundation was made with conventional *in vitro* fertilisation or with intracytoplasmic sperm injection according to medical indication. Fresh or vitrified embryos were used. The embryo transfers were performed upon cleavage status (day 3). ASRM guidelines for the number of embryos to be transferred in the *in vitro* fertilisation cycles were used.

At the time of the embryo transfer, the patients in both groups were put in the lithotomy position. The embryo transfers were performed by 4 medical doctors who specialised in Human Reproduction Biology and were certified by the Mexican Board of Gynecology and Obstetrics and Reproductive Endocrinology. The transfer was guided by an abdominal ultrasound with a full bladder. The cervix was visualised with a vaginal speculum, and then, it was washed with embryo culture medium. Soft catheters were used for embryo transfer (Soft-Pass™ Embryo Transfer Catheter Set, Cook Medical). The soft catheter was charged with 20 μL of embryo culture medium that contained hCG, and the tip of the catheter was placed 15 mm to the fundal limit of the uterine cavity. The embryo culture medium with the hCG preparation included was injected, and the catheter remained for four minutes inside the cavity. Finally, the previously loaded embryos were transferred. In the control group, the same culture media for embryos was used for the transfers but without adding hCG, and it remained for 4 minutes. The pregnancy test with biochemical hCG was performed 12 days after the embryo transfer. If the test was positive, then a transvaginal ultrasound was performed 2 and 4 weeks later, to search for signs of pregnancy, such as the presence of a gestational sac, embryo and fetal heart rate. PROCREA is only a referral centre for reproductive problems. Once a viable pregnancy is confirmed, the patient returns with their gynaecologist for planning their antenatal care.

The biochemical pregnancy was defined as positive from quantitative values of a serum test of β human chorionic gonadotropin according to standard values that are used in the laboratory. The clinical pregnancy rate was defined as a viable pregnancy when there is evidence of a gestational sac, embryo and fetal heart rate at the time of ultrasound evaluation.

Both clínical and biochemical pregnancies are the principal variables that we used when attempting to show the benefits of adding hCG to an altered embryo secretion and damaged endometrium from IVF cycles. The sample size was calculated to compare two proportions. Clinical pregnancy rates of 35% among women in IVF cycles were taken from ASRM reports. We expected to detect an 18% increase in the clinical pregnancy rate, which is 3% higher than the outcomes published by Mansour and Cols. A unilateral test was calculated, and 93 women per group were necessary to obtain a power of 80% at a significance level of 0.05. We used SPSS version 19.0 (IBM) to perform the statistical analysis. We characterised sociodemographic variables with descriptive statistics, using the mean with the standard deviation for the quantitative variables and proportions for the qualitative variables. The student’s *t*-test and Mann–Whitney, where appropriate, were used. For categorical data, the absolute and relative frequencies were determined, and the relative risks with 95% confidence intervals for the proportions were calculated. The Chi-square test and Yates’s correction was used to compare differences in the proportions of both groups. A p < .05 was considered to be statistically significant, to represent significant associations.

## Results

A total of 210 patients were enrolled from August 2011 through November 2012. Table 1 summarises the baseline characteristics of the patients. The intervention group had a patient median age of 36 years, which is one year less than the control group, but no statistically significant differences were found between the groups in terms of the baseline characteristics. IVF with fresh embryos from non-donors represented more than 50% of the patients. Almost 3 out of 10 patients had a previous IVF failed cycle. The fertilisation rate per retrieved oocyte and the number of embryos transferred was similar in both groups. The increases in the implantation and clinical pregnancy rates in the intervention group were statistically significant with a 15% increase in the rates.

**Table 1:** Baseline characteristics

	**Control group**	**Intervention group hCG**	**P value**
	**n 109**	**n 101**	
*Age in years (mean _ SD, range)*	7.3 ± 4.0	36.4 ± 4.5	.151
*Body mass index (mean _ SD, range)*	26.9 ± 2.9	27.1 ± 2.7	.572
*Infertility duration years (mean _ SD, range)*	3.5 ± 1.6	3.3 ± 1.6	.578
*Previous IVF cycles*	32 (29.3%)	28 (27.7%)	.974
*Number of Oocytes retrieved (mean _ SD, range)*	9.6 ± 7.3	8.9 ± 5.0	.483
*Number of Fertilised oocytes (mean _ SD, range)*	6.6 ± 3.6	6.3 ± 3.3	.594
***Infertility types***			
*Female*	32 (29.3%)	28 (27.7%)	
*Male*	22 (20.1%)	19 (18.8%)	
*Mixed*	42 (38.5%)	44 (43.5%)	
*Idiopathic*	13 (11.9%)	10 (9.9%)	
***Technique***			
*IVF*	67 (61.5%)	53 (52.5%)	
*ICSI*	37 (33.9%)	39 (38.6%)	
*PICSI*	5 (4.6%)	9 (8.9%)	
***Types of embryos***			
*Fresh embryos from non-donor*	43 (39.4%)	53 (52.5%)	
*Thawed embryos from non-donor*	30 (27.5%)	25 (24.8%)	
*Donor fresh embryos*	29 (26.6%)	17 (16.8%)	
*Donor thawed embryos*	4 (3.7%)	1 (1.0%)	
*Thawed oocytes from non-donor*	3 (2.8%)	5 (5.0%)	
*Fertilisation rate per retrieved oocytes*	74.8% ± 19.2	75.1% ± 18.9	.931
*Embryos transferred (mean _ SD, range)*	2.1 ± 0.6	2.1 ± 0.5	.469

*ICSI* intra citoplasmic sperm injection. *IVF In vitro* fertilisation. *PICSI* physiological ICSI.

The biochemical pregnancy rate has a relative risk of 1.46, and the clinical pregnancy rate has a relative risk of 1.52. Both of the relative risks calculated have 95% confidence intervals, with statistical significance in the size effect, as we describe in Table 2.

**Table 2:** **Human chorionic gonadotropin (hCG) injected during embryo transfer compared to no injection for infertile women undergoing *in vitro fertilisation cycles***

	**Intervention group (hCG) n/N**	**Control group n/N**	**Relative risk**	**95% confidence intervals**
Biochemical pregnancies	53/101	39/109	1.46	1.07 to 2.00
				p 0.01
Clinical pregnancies	51/101	36/109	1.52	1.09 to 2.12
				p 0.01

*n* outcome; *N* number of participants.

## Discussion

In this study, we confirmed the benefit of intrauterine injection of 500 UI hCG before embryo transfer. The methodology of the study is not the best design for clinical trials to evaluate clinical interventions; however, the evidence has been shown for outcomes in which the clinical pregnancy rate and implantation rate increased significantly in patients in the intervention group. These procedures could benefit all of the patient candidates for IVF/ICSI cycles. The benefit of an early hCG secretion on the embryo before its implantation has been shown [5,6,8-12]. Previous studies have shown that there is a key role for hCG in regulating the inflammatory response and angiogenesis during embryo implantation [12], and an altered damaged endometrial receptivity by the IVF treatments can be overcome by injecting hCG prior to the embryo transfers.

Xiao-Yan et al. showed that embryos in IVF cycles that have a higher secretion of hCG in their culture media have a positive correlation with the implantation rate [13]. We hypothesised that there is an unknown cause that could affect these secretory functions on embryos in the laboratory during the IVF process. Unfortunately, our observation is limited to between 7 and 9 weeks of gestation, which is the time when our team evaluates the viability of the pregnancy through ultrasound. However, miscarriages appear to not be increased in the intervention group before this time, as we describe in Table 3.

**Table 3:** Pregnancy outcomes

	**Control group**	**Intervention group hCG**	**P value**
	**n 109**	**n 101**	
*Biochemical pregnancies (media, proportion)*	39	53	.014
	35.7%	52.4%	
*Clinical pregnancies (media, proportion)*	36	51	.010
	33.0%	50.4%	

This study included patients with a history of recurrent miscarriage and implantation failure. This finding is in contrast with a previous study performed by Mansour et al. in which the first-time IVF patients were enrolled [14]. Selection biases that could affect our internal validation are variables such as age, which were not controlled. The limit was that all patients had to be under 40 years old. The ovarian stimulation protocols were different between the patients according to their particular characteristics. The embryo transfer technique was standardised. The procedure was performed by 4 different operators, and this number of operators could affect our results because it has been demonstrated that embryo transfer techniques differ between operators. Our study also included thawed embryo transfers, in which the benefits of hCG injection were also shown in this group of patients. We did not find complications such as embryo retention in catheters or ectopic pregnancies.

The original methodology was designed to analyse the reproducibility of the results published by Mansour and Cols, which is important given that this study showed significant effects on the pregnancy rates [14]. Our results are similar to those previously reported. One difference to emphasise is that our population group included patients with previous failed IVF cycles and thawed embryos. We hypothesised that early secretion of hCG from the embryo would be decreased before its implantation. This reduction could be for an unknown cause in relation to the laboratory fertilisation or vitrification process. Clinical pregnancy rates in thawed embryo transfers were not our primary outcome, and the statistical analyses were not designed for this approach; however, we observed an improvement in the clinical pregnancy rate, even though the global clinical pregnancy rates are reported to be lower in comparison with reports on fresh embryos.

The reason that we included these thawed embryos is because we hypothesised that the production of hCG by thawed embryos is decreased prior to the implantation process, which is, for some reason, related to the vitrification process. This group of thawed embryos also had benefits in terms of clinical and biochemical pregnancies that resulted from adding the hCG. Because of these results and the previous findings about there being a benefit to endometrium angiogenesis and the inflammatory response, both mechanisms reflect the effects of hCG directly in the endometrium instead of having an autocrine effect on human embryos. More studies with a specific design, such as independent comparisons of fresh and thawed embryo cycles, are needed to elucidate these results in this specific group of patients.

PROCREA is only a referral centre for reproductive problems, and this aspect is a weakness of our study because the follow-up and control of the patients is not possible once the patients return to their gynaecologist for planning their antenatal care. The ultrasound was performed 2–4 weeks after the initial positive serum beta-hCG level. In such cases, the gestational age ranges were 6 to 9 weeks.

The intrauterine injection of hCG before the embryo transfer is a simple procedure. This method does not require complex training and does not consume additional time for the embryologist and clinical staff. No special equipment is necessary for the dilution and preparation of the hCG, and it is not expensive. The intervention offers a benefit to a large heterogeneous group of patients, including those with thawed embryo transfers, which currently represents one third of the live birth rate according to the reports of ASRM/SART [3]. These interventions could be replicated by all IVF units without great effort and could be considered to be without major risk.

## Conclusions

The intrauterine injection of hCG before embryo transfer showed a significant increase in the clinical pregnancy rate. More clinical trials are needed to reproduce these results for this promising intervention. The live birth rate must be included in subsequent studies.

## Abbreviations

ASRM: American Society of Reproductive Medicine; CPR: Clinical pregnancy rate; hCG: Human chorionic gonadotropin; ICSI: Intracytoplasmic sperm injection; IVF: *In vitro* fertilisation; SART: Society for Assisted Reproductive Technology.

## Competing interests

The authors declare that they have no competing interests.

## Authors’ contributions

The conception and the design of the study was performed by ASM and JGV. ASM, OP, APSS, JFJD and OML participated in the selection of the patients and the acquisition of clinical data. OP prepared the catheter with 20 μL of embryo culture medium that contained hCG. JGV and GCB elaborated the methods, interpreted data and did the statistical analysis. All authors read and approved the final manuscript.

## Author information

ASM, JGV, OCL, GCB, APSS and JFJD are Clinical physicians who specialises in Reproductive Medicine and are certified by the Mexican Board of Gynecology and Obstetrics and they are members of the clinical staff of PROCREA. ASM is the medical director of PROCREA. APSS is the president of the ethical committee of PROCREA. JGV and GCB have a master degree in medical sciences. OP is the Embryologist of PROCREA. ASM curently is the secretary of the Mexican Association of Reproductive Medicine.
